# Trauma-induced heme release increases susceptibility to bacterial infection

**DOI:** 10.1172/jci.insight.150813

**Published:** 2021-10-22

**Authors:** Ghee Rye Lee, David Gallo, Rodrigo W. Alves de Souza, Shilpa Tiwari-Heckler, Eva Csizmadia, James D. Harbison, Sidharth Shankar, Valerie Banner-Goodspeed, Michael B. Yaffe, Maria Serena Longhi, Carl J. Hauser, Leo E. Otterbein

**Affiliations:** 1 Department of Surgery and; 2 Anesthesia, Beth Israel Deaconess Medical Center, Harvard Medical School, Boston, Massachusetts, USA.; 3 Koch Institute for Integrative Cancer Research, Massachusetts Institute of Technology, Cambridge, Massachusetts, USA.

**Keywords:** Infectious disease, Inflammation, Bacterial infections, Neutrophils, Translation

## Abstract

Infection is a common complication of major trauma that causes significantly increased morbidity and mortality. The mechanisms, however, linking tissue injury to increased susceptibility to infection remain poorly understood. To study this relationship, we present a potentially novel murine model in which a major liver crush injury is followed by bacterial inoculation into the lung. We find that such tissue trauma both impaired bacterial clearance and was associated with significant elevations in plasma heme levels. While neutrophil (PMN) recruitment to the lung in response to *Staphylococcus aureus* was unchanged after trauma, PMN cleared bacteria poorly. Moreover, PMN show > 50% less expression of TLR2, which is responsible, in part, for bacterial recognition. Administration of heme effectively substituted for trauma. Finally, day 1 trauma patients (*n* = 9) showed similar elevations in free heme compared with that seen after murine liver injury, and circulating PMN showed similar TLR2 reduction compared with volunteers (*n* = 6). These findings correlate to high infection rates.

## Graphical abstract

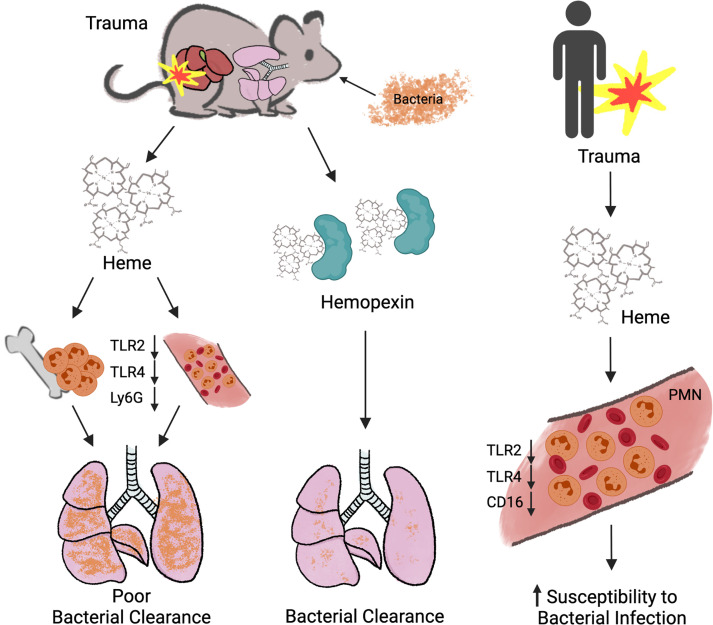

## Introduction

Trauma-related injuries are the third leading cause of death in the United States and the most common cause of death in persons younger than 45 (1). Moreover, these rates are continuing to rise: from 2000 to 2010, the number of trauma deaths increased by 23% (2), while death rates for cancer and heart disease declined by 20% between 1991 and 2009 (3) and 31% between 2000 and 2010 (4). Importantly, nosocomial infection and multiple organ failure (MOF) remain the major causes of late mortality in trauma patients (5, 6). While brain injury and hemorrhage lead to early deaths, with a median time of less than 24 hours, nosocomial infections and MOF contribute to deaths that occur days or weeks later (7, 8). Thus, trauma patients who survive their initial injuries have a significantly heightened susceptibility to infection at sites remote from the primary injury that places them at risk for mortality (9). In efforts to align human pathology of trauma and increased infection rates, we developed a 2-hit model in mice that is composed of a liver crush injury, followed by a bacterial inoculation of the lung.

The dominant mechanism by which the injured host is thought to become susceptible to infection involves the effects of damage-associated molecular pattern (DAMP) molecules released and accumulated as a result of tissue injury. These include purine metabolites, S100 family members, HMGB1, and heme (10–12). In addition, as evolutionary endosymbionts, mitochondria bear close molecular similarities to bacterial pathogen associated molecular patterns (PAMPs) that, like DAMPs, can trigger powerful innate immune responses (13). Collectively, DAMPs and PAMPs are potent immunomodulators, and cells have evolved elegant cognate receptors and signaling cascades by which these molecules modulate cell function. DAMPs and PAMPs induce the prototypical cytokine storm, oxidant radical generation, transcription factor mobilization, and resulting stress response gene expression (12). Paradoxically, this early sequence of events can also lead to immunosuppressive events and can place the host at an increased risk of infection by bacteria that would be successfully cleared by an archetypical myeloid cell response absent the traumatic injury.

Heme is a complex of iron and protoporphyrin IX that serves multiple essential functions in aerobic organisms as the prosthetic group of hemoproteins. Key examples include hemoglobin and myoglobin, the oxidases of the mitochondrial electron transport chain, cytochrome p450, and other signaling proteins like guanylate cyclase and the nitric oxide synthases (14). Under normal conditions, heme is critical to normal enzymatic function. In contrast, elevated levels of free heme occurring at times of cell and tissue injury or during hemolysis can be toxic due to their ability to elicit oxidative stress and inflammation. Fortunately, robust systems are in place that clear extracellular free heme. These begin with a battery of serum heme-binding proteins that include hemopexin (Hpx), haptoglobin, and albumin. However, heme can also bind to members of the Toll family of receptors, including TLR2 and TLR4 (15–17). When these receptors bind heme, they can activate NF-κB–dependent proinflammatory signaling events. Free heme is ultimately cleared by transport into the cell, where it is rapidly metabolized by Heme Oxygenase 1 (HO-1) and HO-2 (gene *Hmox*) (16, 18, 19).

One of the common bacterial strains causing nosocomial infection after trauma is the Gram^+^ bacterium *Staphylococcus aureus* (*S*. *aureus*) (20–22), which is normally an innocuous colonizer of the nose and skin in healthy humans. When the host immune system is compromised by injury, however, *S*. *aureus* can become an important infective organism (23, 24). We developed this posttraumatic pneumonia model as a translational research tool to study the pathophysiology and mechanisms of immune susceptibility to opportunistic infection following traumatic organ injury. Our objective was to better understand how an initiating tissue injury–induced release of heme results in alterations in the host’s response to a second challenge of bacterial inoculation in the lung. We focused on the neutrophil as the primary cell that infiltrates to sites of infection and identify a population of neutrophils (PMN) that, while recruited normally, are poor at clearing bacteria.

## Results

### Trauma to the liver impairs subsequent bacterial clearance in the lung.

**Figure 1:**
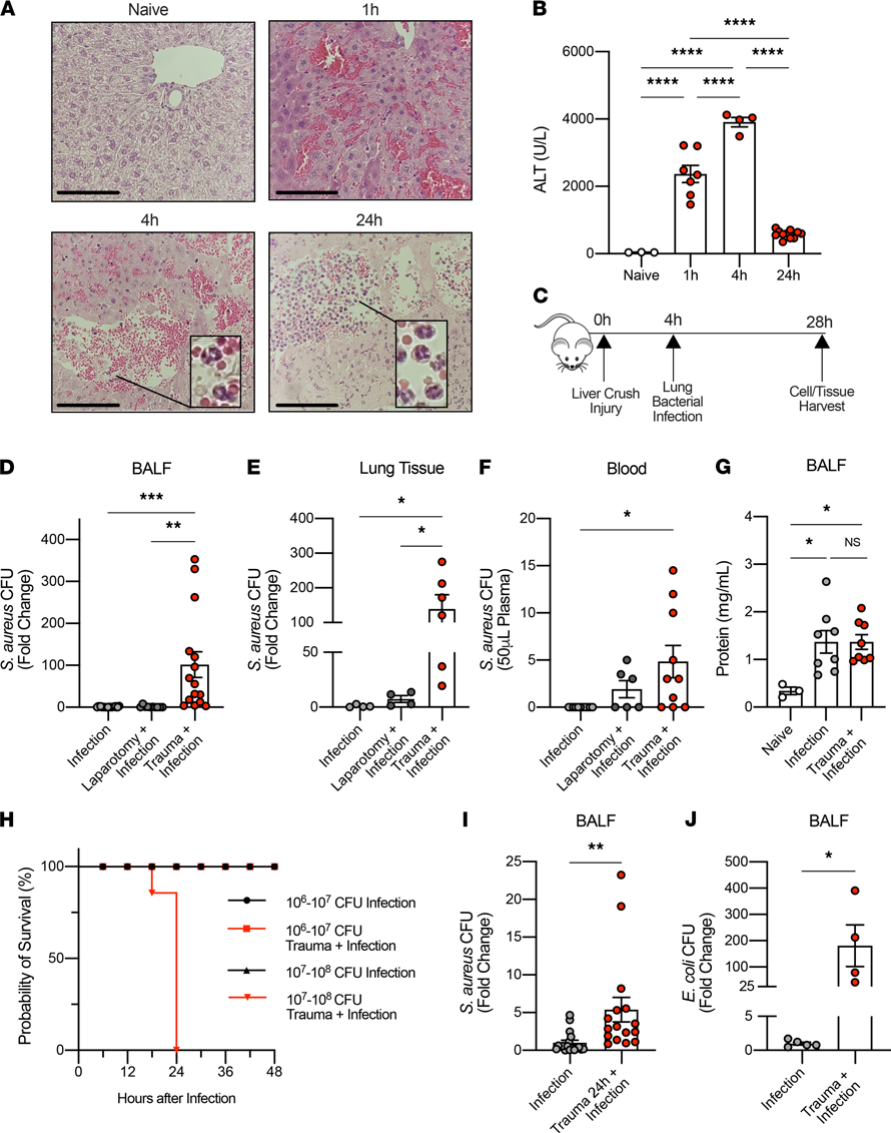
Liver crush injury decreases bacterial clearance in the lung. (**A**) Representative images of the naive left liver lobe and 1, 4, and 24 hours after injury (5 sections/tissue, *n* = 3/group). Scale bar: 100 μm. Original magnification, ×200. Close-up images of PMN are shown. (**B**) Levels of alanine aminotransferase (ALT) in plasma before (*n* = 3) and after liver crush injury (*n* = 4-11/group). (**C**) Posttraumatic pneumonia model: liver crush injury is performed in the left liver lobe, and 4 hours later, mice are challenged with 1 × 10^6^ to 1 × 10^7^ CFU of *S*. *aureus* in the lung. Twenty-four hours after infection, cells and tissues were harvested for further analysis. (**D** and **E**) Fold change of *S*. *aureus* CFU in bronchoalveolar fluid (BALF) after infection only, after infection with laparotomy or liver crush injury (*n* = 10–19/group) and in lung tissues (*n* = 4–6/group). (**F**) *S*. *aureus* CFU in 50 μL of plasma in the post-traumatic pneumonia model (*n* = 6–10/group). (**G**) Protein levels in the BALF at the baseline (*n* = 3), 24 hours after infection with and without liver crush injury (*n* = 8/group). (**H**) Survival curve of mice with and without liver crush and with 1 × 10^6^ to 1 × 10^7^ or 1 × 10^7^ to 1 × 10^8^ CFU of *S*. *aureus* (*n* = 4–5/group). (**I**) Fold change of *S*. *aureus* CFU in BALF after infection only or with liver crush injury performed 24 hours prior to the infection (*n* = 18–20/group). (**J**) Fold change of *E*. *coli* CFU in BALF collected 24 hours after infection, with and without liver crush injury performed 4 hours prior to the infection (*n* = 4–5/group). All data are presented as mean ± SEM. Statistical analyses for **I** and **J** were performed by unpaired 2-tailed Student’s *t* test. All other *P* values were calculated by 1-way ANOVA with post hoc Tukey’s test. *P < 0.05, **P < 0.01, ***P < 0.001, ****P < 0.0001.

Trauma patients have a higher susceptibility to develop infection (9), and little is known about the mechanism by which sterile injuries can heighten a subjects’ susceptibility to pathogens that are usually benign in tissues distant from the injured site. Since liver is the solid organ that is damaged most commonly as a result of blunt trauma (25–27), we developed and standardized a model of nonlethal blunt liver trauma in mice. One hour after liver crush, clotting was observed both macroscopically (not shown) and microscopically (Figure 1A). Four hours after liver crush, PMN rose in the circulation and accumulate in the injured liver (Figure 1A and Supplemental Figure 1, A–C; supplemental material available online with this article; https://doi.org/10.1172/jci.insight.150813DS1). Standardization of the severity of the crush injury was confirmed by measuring serum alanine aminotransferase (ALT) levels over time (Figure 1B).

To test whether liver injury alters host bacterial clearance from the lung, mice were inoculated with 1 × 10^6^ to 1 × 10^7^ CFU of *S*. *aureus* into the lungs 4 hours after liver crush injury, as depicted in Figure 1C. *S*. *aureus* is a clinically relevant pathogen that commonly causes early posttraumatic pneumonia in at-risk trauma subjects (6). Twenty-four hours after inoculation, animals were euthanized ,and a bronchoalveolar lavage (BAL) was performed to measure cell and bacteria counts. Mice without surgery and mice with only laparotomy cleared bacteria effectively from the lung at 24 hours. Mice subjected to liver crush, however, showed 100-fold more bacteria in the BAL and the lung tissues at 24 hours when compared with sham controls (Figure 1, D and E). Similarly, higher *S*. *aureus* counts were detected in the blood of infected mice with liver crush injury (Figure 1F). Lung-infected mice showed increased accumulation of protein in the BAL as a marker of lung injury, but no significant differences were observed between infected mice with and without liver injury (Figure 1G). However, a greater level of inflammation was observed by blinded histological analysis in crush plus infection compared with infection alone (Supplemental Figure 2A). Mice with liver injury alone showed no translocation of endogenous bacteria into the blood, lung tissue, or BAL fluid (data not shown). All mice survived liver crush injury plus infection, with bacterial clearance observed 48 hours after inoculation in both groups (Supplemental Figure 2B). However, mice challenged with a higher dose of *S*. *aureus* in the presence of liver crush all died, while all survived the high-dose inoculation in the absence of liver crush (Figure 1H).

When bacterial inoculation was delayed 24 hours after trauma, we found similar deficiencies in animals’ ability to clear bacteria as compared with controls (Figure 1I). We also noted that liver injury had the same effect on Gram^–^ *E*. *coli* clearance as *S*. *aureus* (Figure 1J). Taken together, the results show that an increased susceptibility to bacterial infection similar to that observed in human trauma can be simulated by liver trauma in mice.

### Trauma injury results in a transient release of free heme in mice and humans.

**Figure 2:**
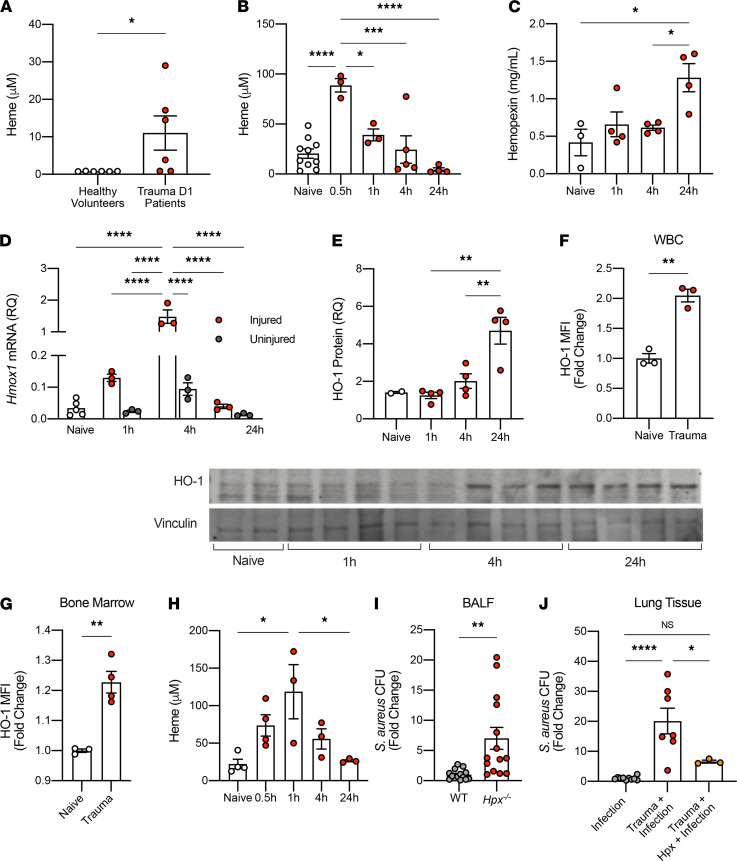
Trauma injury releases free heme. (**A**) Levels of free heme in the plasma from healthy volunteers and trauma patients on day 1 (*n* = 6/group). (**B** and **C**) Levels of free heme (*n* = 3–10/group) and hemopexin (Hpx; *n* = 3–4/group) in the plasma after liver crush injury in mice. (**D**) *Hmox1* mRNA expression levels in the injured left liver lobes and the uninjured right liver lobes after crush injury (normalized to beta-actin; *n* = 3–5/group). (**E**) HO-1 protein levels in the naive (*n* = 2) and injured liver lobes after crush injury (normalized to vinculin; *n* = 4/group). Western blot images are shown below. (**F** and **G**) Mean fluorescent intensity (MFI) of HO-1 in blood cells (WBC; *n* = 3/group) and BM cells 24 hours after liver crush injury (*n* = 4/group). (**H**) Levels of free heme in the plasma after liver crush injury in *Hpx^–/–^* mice (*n* = 3-4/group). (**I**) Fold change of *S*. *aureus* CFU in the BALF 24 hours after infection in C57BL/6 mice and *Hpx^–/–^* mice, both with liver crush injury performed 4 hours before the infection (*n* = 14/group). (**J**) Fold change of *S*. *aureus* CFU in the lungs 24 hours after infection in C57BL/6 mice with infection only (*n* = 11), trauma followed by infection (*n* = 7), and trauma followed by Hpx treatment and then infection (*n* = 3). All data are presented as mean ± SEM. Statistical analyses for **A**, **F**, **G**, and **I** were performed by unpaired 2-tailed Student’s *t* test. All other *P* values were calculated by 1-way ANOVA with post hoc Tukey’s test. *P < 0.05, **P < 0.01, ***P < 0.001, ****P < 0.0001.

Traumatic injury can lead to bleeding with release of heme from extravascular blood, as well as from the intracellular contents of damaged or dying cells. Under these circumstances, heme can act as a potent DAMP and can contribute to further cellular injury (12). Healthy humans have very low or undetectable levels of free heme in the plasma (<1 μM, Figure 2A). In contrast, plasma samples from trauma patients collected 1 day after traumatic injury showed a 10-fold rise in free serum heme (Figure 2A). Variability may reflect the time the first blood sample was collected relative to the time the trauma occurred. Similarly, mice subjected to a liver crush injury showed a time-dependent rise in circulating free heme from a baseline of 25 μM that peaked at > 75 μM within 30 minutes after injury and returned to baseline by 4 hours (Figure 2B). The difference in peak heme levels between humans and mice may be attributed to the type of injury and/or differences in physiological and metabolic response times between humans and mice. Given the unpredictability of human trauma, it is challenging to generate a specific kinetic of heme release in trauma patients; thus, we grouped plasma collected from trauma patients into batches. It is likely that peak plasma heme levels occur early after injury depending on the severity and cause. Hpx is a serum protein whose principal role is to scavenge and bind free heme. After liver crush, Hpx levels in plasma increased and peaked at 24 hours (Figure 2C). Elevated Hpx levels most likely reflect an acute phase mechanism to clear free heme and, thus, limit its activity as a DAMP. Measurement of plasma Hpx in patients collected on days 0, 1, and 3 after trauma showed no significant differences compared with healthy volunteers (data not shown). Explanations might include timing of the collection of the blood samples, the small sample size, or a difference between trauma in mice and trauma in humans.

Heme is well known to be metabolized by heme oxygenases. Therefore, next, we measured inducible HO-1 as a response to the increase in free heme elicited by liver crush injury. HO-1 expression increased in both the injured and uninjured liver lobes at 4 hours at the mRNA level, but it was significantly higher in the injured lobe (Figure 2D). HO-1 protein expression peaked at 24 hours in the injured liver lobes (Figure 2E). Additionally, both circulating WBC and BM PMN showed increased HO-1 expression 24 hours after liver crush (Figure 2, F and G). These data suggest that heme, rapidly released as a result of tissue injury, is taken up and processed at multiple sites based on HO-1 expression. Of note, acute inflammation will also increase HO-1 independently of free heme (28).

Based on the serum heme data, we next tested the hypothesis that free heme might be responsible, at least in part, for the immune dysfunction observed in the lung in response to bacteria after trauma. To test this, we performed liver crush injury plus infection in *Hpx^–/–^* mice. *Hpx^–/–^* animals showed a similar rise in plasma heme levels, but heme clearance was delayed, peaking at 1 hour compared with 30 minutes in WT controls (Figure 2, B and H). Free heme is eventually cleared from the circulation after trauma in *Hpx^–/–^* animals, likely by the secondary scavengers haptoglobin and albumin. Notably, however, *Hpx^–/–^* mice were far worse at clearing bacteria from the lung after liver crush than were WT injury-plus-infection mice (Figure 2I).

We also tested whether scavenging free heme released after liver crush by Hpx would reverse the effect of liver crush injury on the increased host susceptibility to bacterial infection. To test this, Hpx was administered to mice twice after liver crush injury, shortly after the injury (50 mg/kg, i.v. in PBS), and again 45 minutes after the injury (50 mg/kg, i.p. in PBS). Injured mice, inoculated with bacteria and treated with Hpx, showed that mice cleared bacteria to levels equivalent to infected mice without a liver crush injury (Figure 2J). Collectively, the data support a significant causative role for heme released by traumatic injury in the observed impairment of bacterial clearance.

### Traumatic injury does not affect recruitment of PMN into the lung in response to bacterial infection.

**Figure 3:**
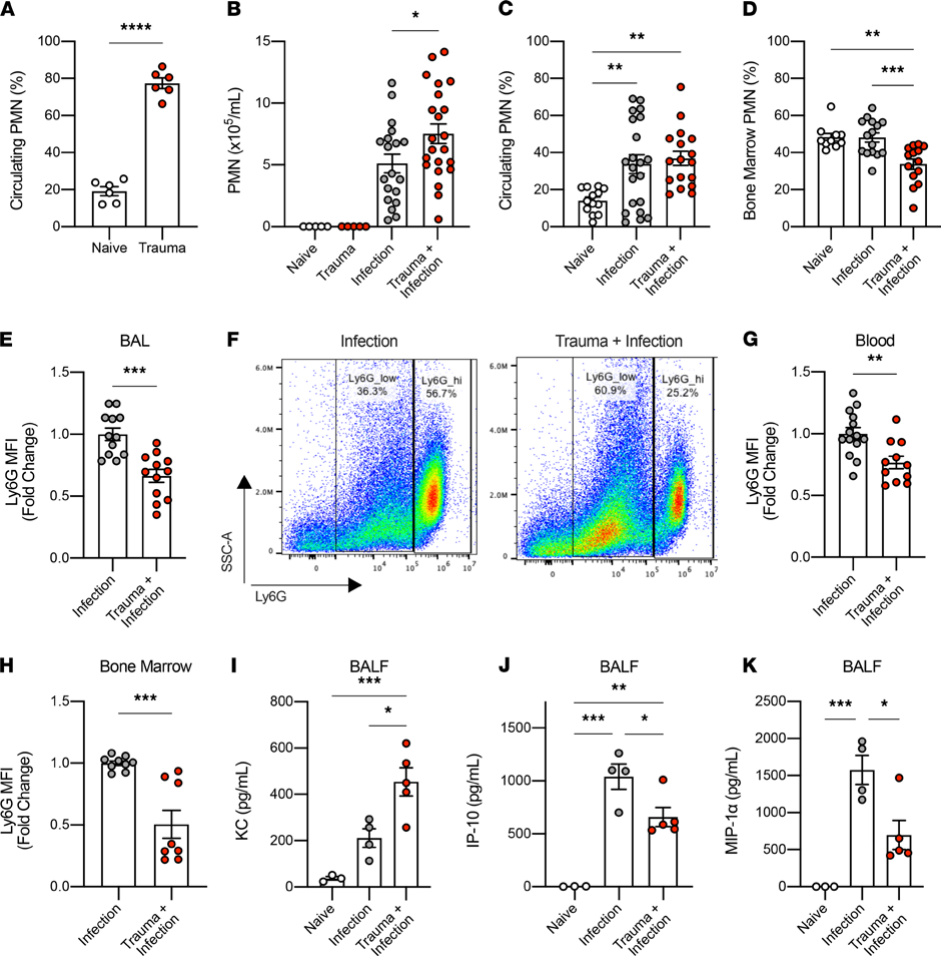
Liver crush injury does not impair PMN migration to the sites of bacterial infection. (**A**) Frequency of CD11b^+^Ly6G^+^ neutrophils (PMN) in the blood 4 hours after liver crush injury (*n* = 6/group). (**B**) Absolute number of PMN in 1 mL of BALF at the baseline (*n* = 5), 4 hours after liver crush injury alone (*n* = 5), 24 hours after lung infection with and without liver crush injury (*n* = 20/group). (**C** and **D**) Frequency of PMN in the blood (*n* = 13–21/group) and the BM (*n* = 10–15/group), 24 hours after infection with and without liver crush injury. (**E**) Fold change of Ly6G MFI in PMN in BAL after lung infection with and without liver crush injury (*n* = 12/group). (**F**) A representative flow cytometry analysis image showing the MFI of Ly6G in PMN in BAL (left, infection only; right, trauma + infection). (**G** and **H**) Fold change of Ly6G MFI in PMN in the blood (*n* = 11–14/group) and the BM (*n* = 8–9/group) 24 hours after infection, with and without liver crush injury. (**I**–**K**) Levels of KC, IP-10, and MIP-1α in the BALF at the baseline (*n* = 3), 24 hours after infection with and without liver crush injury (*n* = 4–5/group). All data are presented as mean ± SEM. Statistical analyses for **A**, **E**, **G**, and **H** were performed by unpaired 2-tailed Student’s *t* test. All other *P* values were calculated by 1-way ANOVA with post hoc Tukey’s test. *P < 0.05, **P < 0.01, ***P < 0.001, ****P < 0.0001.

A significant increase in the number of circulating PMN was noted 4 hours after liver crush injury, many of which are Ly6G^+^ (Figure 3A and Supplemental Figure 1C). Of note, no PMN were observed in the BAL in mice with liver crush injury alone (Figure 3B). Since PMN are also the first cells recruited to sites of infection, we posited that the lack of bacterial clearance after trauma might simply be due to insufficient PMN recruitment into the airway to kill bacteria. We found, however, that PMN migration was unaffected by crush injury (Figure 3B). Analysis of circulating and BM PMN showed a comparable percentage of PMN in peripheral blood and a significantly lower percentage of PMN in the BM when compared with PMN from infection without trauma (Figure 3, C and D). Remarkably, PMN recruited to the airways after infection exhibited much lower Ly6G expression levels in the mice with liver crush injury compared with the mice without the injury (Figure 3, E and F). Profiling of the Ly6G^+^CD11b^+^ populations in peripheral blood and BM showed that both circulating and BM PMN exhibited low Ly6G expression (Figure 3, G and H), which was similar to that observed in the BAL. These data suggest that more PMN migrated from the BM into the circulation and then into the lung in mice subjected to liver crush injury, followed by bacterial infection. PMN populations that have low expression of Ly6G (Ly6G^lo^) or intermediate expression of Ly6G (Ly6G^int^) have been characterized as immature PMN by others (29, 30). It is possible that the more mature PMN populations are recruited to the liver in response to injury, as PMN with higher Ly6G expression, indicative of more mature PMN, was observed in the circulation 4 hours after liver injury alone (Supplemental Figure 1C); thus, a concurrent immune challenge such as bacterial inoculation at a remote site may show a greater propensity to recruit immature PMN to the site since there would be insufficient mature PMN available. Again, this might contribute to infective risk.

The levels of keratinocyte-derived chemokine (KC; CXCL1), a potent chemoattractant for PMN to the sites of infection, were significantly higher in the BAL fluid in mice with liver crush injury compared with naive mice and mice with infection (Figure 3I), which may explain in part the enhanced migration of PMN into the lung in mice subjected to trauma before bacterial inoculation. Similar upregulation of KC after traumatic injuries has been reported by others (31). Additionally, we measured the levels of IFN-γ–induced protein 10 (IP-10; CXCL10) and macrophage inflammatory protein-1α (MIP-1α; CCL3) in BAL fluid. These cytokines are known to be released by PMN and are implicated in bacterial killing and clearance (32, 33). Interestingly, significantly lower amounts of IP-10 and MIP-1α were detected in infected mice after liver crush injury (Figure 3, J and K). Despite the enhanced recruitment of PMN to the site of lung infection, lower levels of IP-10 and MIP-1α suggest that immature PMN are dysfunctional and unable to contribute to an innate inflammatory response against a bacterial challenge.

### Trauma decreases TLR2 expression in PMN and macrophages.

**Figure 4:**
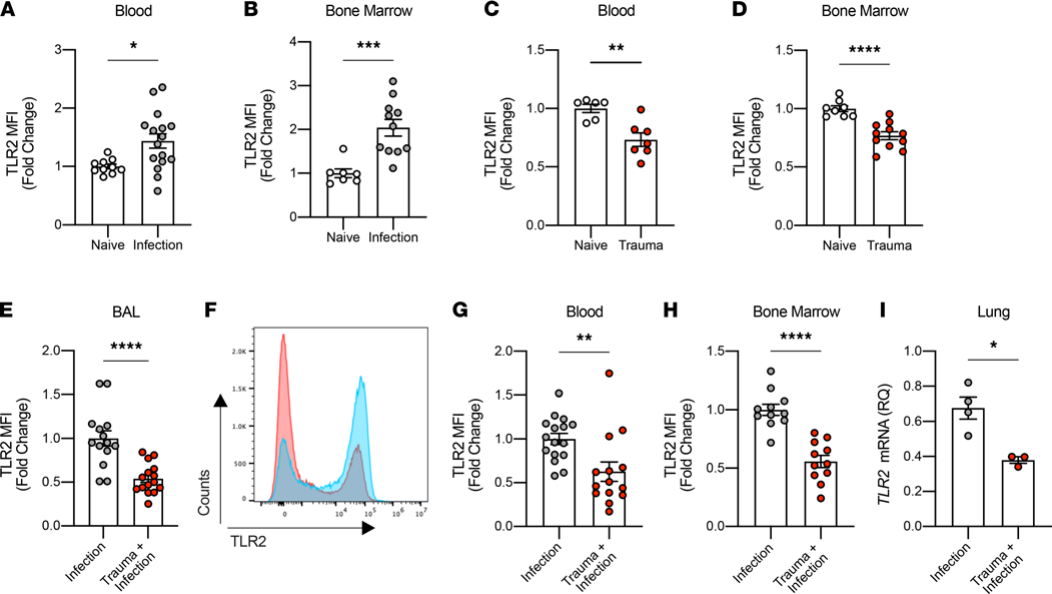
Liver crush injury downregulates TLR2 expression in PMN in vivo. (**A** and **B**) Fold change of TLR2 MFI in PMN 24 hours after *S*. *aureus* lung infection in the blood (*n* = 10–16/group) and the BM (*n* = 7–11/group). (**C** and **D**) Fold change of TLR2 MFI in PMN 4 hours after liver crush injury in the blood (*n* = 6–7/group) and the BM (*n* = 8–11/group). (**E**) Fold change of TLR2 MFI in PMN in BAL, 24 hours after infection with and without liver crush injury (*n* = 14–15/group). (**F**) A representative flow cytometry analysis image showing the MFI of TLR2 in PMN in BAL. Blue represents the PMN in the BAL from the “Infection” group, and red represents the PMN from the “Trauma + Infection” group. (**G** and **H**) Fold change of TLR2 MFI in PMN in the blood (*n* = 14–16/group) and the BM (*n* = 11/group), 24 hours after infection, with and without liver crush injury. (**I**) *TLR2* mRNA expression levels in lung tissues, 24 hours after *S*. *aureus* infection with and without liver crush injury (normalized to *HPRT1* mRNA expression; *n* = 3–4/group). All data are presented as mean ± SEM. Statistical analysis was performed by unpaired 2-tailed Student’s *t* test. *P < 0.05, **P < 0.01, ***P < 0.001, ****P < 0.0001.

TLR2 contributes to appropriate recognition of *S*. *aureus* by binding lipoteichoic acid, a component of the cell wall that functions as a PAMP (34). When this pathogen’s molecules bind TLR2, a signaling cascade results in translocation of NF-κB and cellular activation (35, 36). As predicted, inoculation of the lung with *S*. *aureus* increased TLR2 expression in circulating PMN and BM PMN (Figure 4, A and B). We then tested whether liver crush injury alone altered expression of TLR2. Trauma induced downregulation of TLR2 in PMN in both the blood and BM 4 hours after liver crush (Figure 4, C and D). TLR2 expression was significantly lower in PMN in the BAL after liver crush compared with laparotomy-only plus infection (Figure 4, E and F). This reduction in TLR2 expression after trauma plus infection was also observed in PMN from lung, peripheral blood, and BM (Figure 4, G–I).

PMN that migrate into the airway in response to bacterial infection express lower TLR2 receptor and, therefore, are likely be at a disadvantage in recognizing *S*. *aureus*. Collectively, these data suggest that DAMPs released from crushed liver modify the PMN pool such that the more mature PMN are recruited to the injury site in the liver (Supplemental Figure 1), leaving more immature PMN populations in the circulation and BM that are less capable of clearing bacteria when recruited to the lung.

Alveolar macrophages are also crucial for host defense against pathogen invasion of the lung. The decreased levels of TLR2 after liver crush injury followed by infection are not limited to the PMN, since alveolar macrophages also showed decreased TLR2 expression in infected mice after liver crush when compared with infection in the absence of trauma (Supplemental Figure 3, C and D).

### Heme impairs bacterial clearance in the lung by decreasing TLR2 levels on PMN.

**Figure 5:**
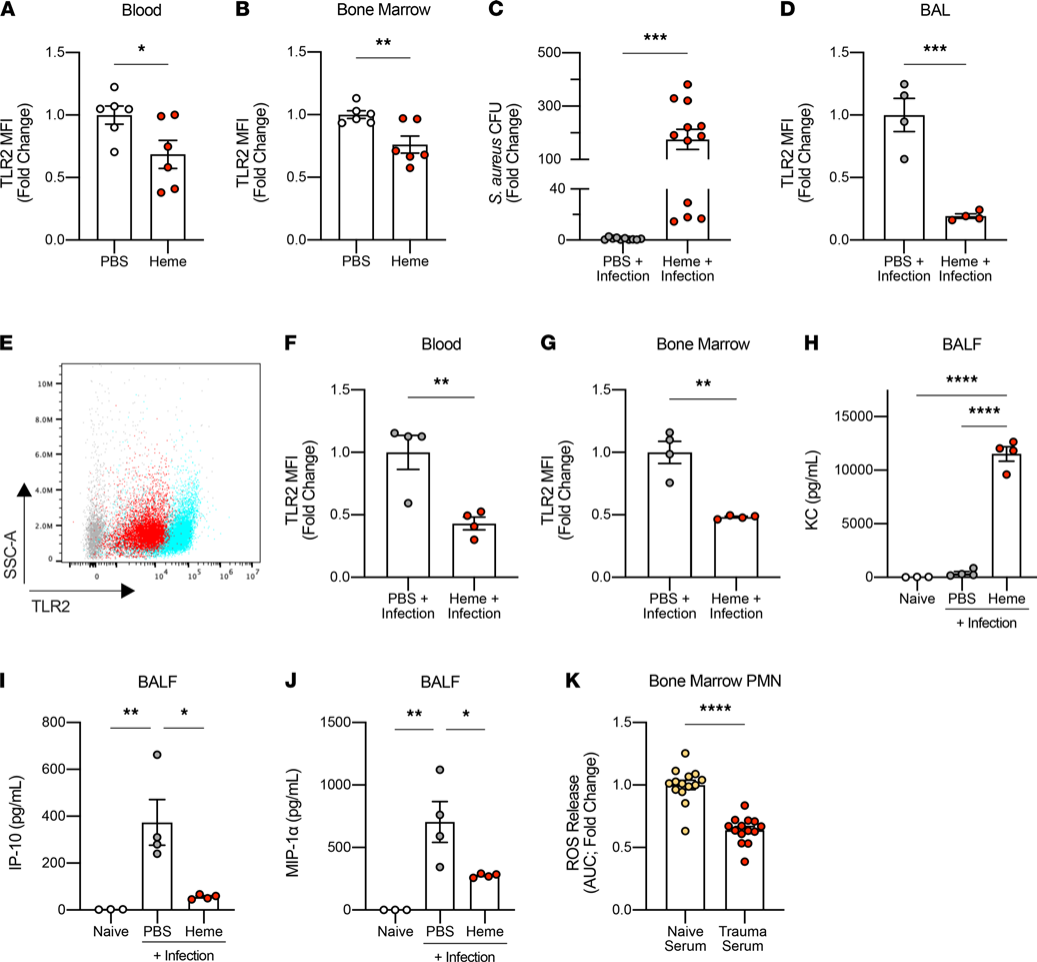
Heme decreases bacterial clearance in the lung and decreases TLR2 expression in PMN in vivo. (**A** and **B**) Fold change of TLR2 MFI in PMN in the blood and the BM, 4 hours after heme challenge alone (50 mg/kg, i.p.) in vivo (*n* = 6/group). (**C**) Fold change of *S*. *aureus* CFU in BALF 24 hours after infection with and without heme challenge. Mice were challenged with heme 4 hours before the infection (50 mg/kg, i.p.; *n* = 10–12/group). (**D**) Fold change of TLR2 MFI in PMN in BAL 24 hours after infection with and without heme challenge (*n* = 4/group). (**E**) A representative flow cytometry analysis image showing the MFI of TLR2 in PMN in the BAL. Blue represents PMN in BAL from control mice, and red represents PMN from mice with heme challenge. Gray represents unstained cells. (**F** and **G**) Fold change of TLR2 MFI in PMN in the blood and the BM 24 hours after infection, with and without heme challenge (*n* = 4/group). (**H**–**J**) Levels of KC, IP-10, and MIP-1α in the BALF at the baseline (*n* = 3), 24 hours after lung infection with and without heme challenge (*n* = 4/group). (**K**) Fold change of reactive oxygen species (ROS) release in PMN treated with naive or trauma serum in response to *S*. *aureus* exposure in vitro (3 independent experiments, each with *n* = 3–4/group). ROS release is measured by the AUC. All data are presented as mean ± SEM. Statistical analyses for **H**, **I**, and **J** were performed by 1-way ANOVA with post hoc Tukey’s test. All other *P* values were calculated by unpaired 2-tailed Student’s *t* test. *P < 0.05, **P < 0.01, ***P < 0.001, ****P < 0.0001.

Heme as a DAMP is known to activate innate immunity both by increasing oxidative stress and by direct binding of TLR4 (12). Based on elevated plasma heme levels after trauma, we next asked whether the downregulation of TLR2 following liver crush involved free heme. To test this, mice were challenged with a sublethal dose of heme (50 mg/kg, i.p.) to mimic release of heme by injured tissues. As seen after liver trauma, mice treated with heme exhibited lower TLR2 expression in circulating PMN and BM PMN (Figure 5, A and B). Furthermore, mice treated with heme followed 4 hours later by *S*. *aureus* inoculation in the lung were unable to effectively clear *S*. *aureus* (Figure 5C). PMN recruited to the lung as well as peripheral blood and BM PMN exhibited significantly lower expression of TLR2 after heme challenge (Figure 5, D–G).

The mechanism by which TLR2 is regulated in response to heme is not known, but heme is a known ligand for TLR4 (16, 17). Thus, we also examined TLR4 expression after heme and in response to liver crush. Similar to TLR2, we observed a significant reduction in TLR4 expression in circulating PMN after liver crush and heme challenge (Supplemental Figure 3, E and F). This decrease in TLR4 expression may also explain, in part, the observed impairment in *E*. *coli* clearance in the lung after liver crush (Figure 1J). These data suggest that liver crush injury, presumably through release of heme, increases susceptibility to bacterial infection after trauma and that this effect is explained, in part, by reduced expression of TLR2 and TLR4. In addition to TLR2 and TLR4, we observed that both TLR1 and TLR5 expression were reduced in BAL PMN (Supplemental Figure 3, A and B). TLR1 is known to form a heterodimer with TLR2 (37). The role of TLR5 in *S*. *aureus* infection is not well known; thus, these findings warrant further investigation. Collectively, these data further support our findings.

Similar to the observation made in mice with lung infection after liver crush injury, heme-challenged mice also exhibited significantly higher levels of KC in the BAL fluid after lung infection (Figure 5H). Furthermore, the levels of IP-10 and MIP-1α were significantly lower in the BAL fluid of infected mice in the presence of heme (Figure 5, I and J). Like the dysfunction of PMN observed after liver crush, these data implicate heme as the DAMP that impairs release of IP-10 and MIP-1α, ultimately preventing bacterial clearance.

We next tested whether the serum collected after liver crush injury when plasma heme levels are peaking would influence PMN antimicrobial function by measuring their ability to generate reactive oxygen species (ROS). To test this, trauma serum was collected 30 minutes after liver crush injury when heme levels peak (Figure 2B). PMN, isolated from the BM of naive mice negatively selected against Ly6G^+^ cells, were then cultured with naive mouse serum or trauma serum for 90 minutes. PMN were then exposed to *S*. *aureus*, and ROS were measured by chemiluminescence as described in Methods. PMN incubated with trauma serum showed reduced ROS generation compared with naive serum–treated PMN, suggesting a potential mechanism that would explain, in part, the lack of bacterial clearance (Figure 5K).

### PMN from trauma patients show reduced expression of CD16, TLR2, and TLR4.

**Figure 6:**
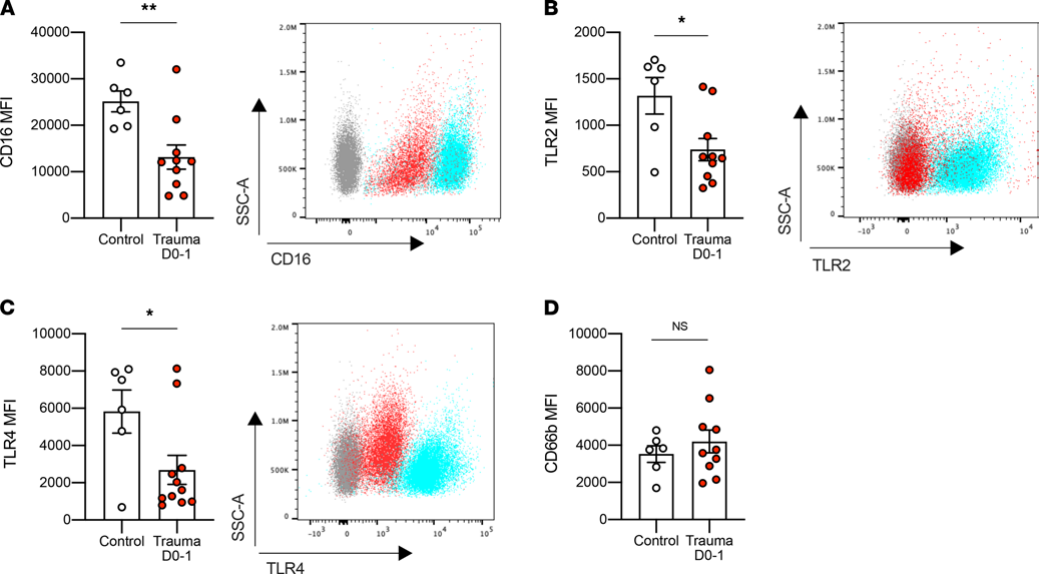
Trauma injury downregulates CD16, TLR2, and TLR4 expression in circulating PMN in patients. (**A**–**D**) MFI of CD16, TLR2, TLR4, and CD66b in circulating PMN collected from control patients (*n* = 6) and trauma patients on day 0 or 1 (*n* = 9) measured by flow cytometry. A representative flow cytometry analysis image is shown on the right (except for CD66b). Gray represents unstained cells, blue the control, and red the trauma. All data are presented as mean ± SEM. Statistical analysis was performed by using unpaired 2-tailed Student’s *t* test. *P < 0.05, **P < 0.01.

We next characterized circulating PMN purified from blood of trauma patients 1 day after major trauma by measuring expression of CD16, CD66b, TLR2, and TLR4. Like Ly6G^lo^ cells in mice, nearly all PMN isolated from peripheral blood in trauma patients were CD16^lo^ or CD16^–^ (Figure 6A) and, therefore, would generally be considered “immature” PMN (38, 39). These findings are consistent with prior reports that showed significantly lower PMN expression of CD16 after major trauma (40). Low expression of Ly6G or CD16 are both considered characteristic of immature PMN (41, 42).

In addition to decreased CD16, circulating PMN from trauma subjects showed lower expression of both TLR2 and TLR4 shortly after injury (Figure 6, B and C). Again, these findings parallel those observed in mice. Human PMN do not express Ly6G, but notably expression of CD66b, another PMN activation marker, showed no differences between PMN from trauma and control subjects (Figure 6D). This shows that the decreases in TLR2, TLR4, and CD16 seen do not reflect global receptor downregulation. Of note, 5 of 9 trauma patients developed infections during their hospital stay (Table 1). Therefore, again, the human data correlate with the mouse model; collectively, the data suggest that downregulation of TLR2 and/or TLR4 as a result of heme released by traumatic injuries may be an important cause of increased clinical susceptibility to bacterial infection.

## Discussion

An individual’s airway is not sterile; rather, constant low-level inoculation from the digestive tract is cleared by innate immune responses that prevent establishment of the inoculum (43–47). After injury, this homeostasis is disturbed, and invasive infection becomes more common. The murine model of posttraumatic pneumonia we developed and characterized for this study closely mimics that aspect of human injury. Inoculation of the lung with a subeffective dose of bacteria in the presence of a liver crush injury recapitulates human pathology, showing how trauma can predispose both animals and humans to bacterial infection. Moreover, we show here for the first time to our knowledge that the mechanism involves, in part, downregulation of PMN TLR2 and TLR4 in both species. Thus, trauma suppresses a primary receptor mechanism used by the host to sense the presence of pathogens, effectively creating an immunosuppressive environment at barrier sites like the lung that may be distant from physical injuries. Cahill et al. employed a multiplex mediator signature to differentiate sepsis from sterile systemic inflammatory response syndrome in humans and concluded that biomarker release patterns can be used as powerful diagnostics that, in turn, direct personalized treatment strategies (48). Applied here, patterns of TLR expression in conjunction with specific cytokine/chemokine expression could be used to determine susceptibility to infection.

In fact, we see here that trauma causes multiple phenotypic changes in innate immune cells, particularly PMN, since those cells are sequentially mobilized into the circulation and then to injury sites. Those events appear to markedly alter the phenotype of cells available to respond to distant infections. In response to a liver crush injury, a significant number of PMN is mobilized to the liver as a prototypic response to tissue injury. Thus, we initially speculated that impaired lung bacterial clearance after injury might reflect either a transient neutropenia that limited the number of cells available for recruitment, or reflect a defect in PMN migration. Trauma did not, however, impair PMN migration to sites of bacterial infection in this model, but it did modify alveolar macrophages. We observed a modest but significant increase in PMN presence in the airway in response to bacteria. The enhanced PMN recruitment to the site of infection is likely due, in part, to the greater amounts of KC in the BAL fluid (Figure 3I). What is intriguing is that this does not ensure that infiltrating PMN are in fact functional. The PMN that migrated exhibited low Ly6G expression, a phenotype of immature PMN (or “bands”) (29, 30, 41, 42). This population of cells is thought to be sequestered from the BM prematurely but still transmigrates into the airway in response to chemokine gradients generated in response to bacteria inoculation (49). Circulating human PMN after injury showed low CD16 expression, similarly considered a marker of immature PMN (40). Emergence of immature granulocytes in trauma patients has been documented by others, and perhaps these populations of PMN are phenotypically and functionally different (40, 50, 51). Indeed, there are N1 and N2 PMN that exhibit different functionality — N1 being proinflammatory and N2 being antiinflammatory (52, 53). Neely et al. have demonstrated that, in a murine model of burn injury with infection, N1 PMN (IL-10^–^IL-12^+^) are more effective than N2 PMN (IL-10^+^IL-12^–^) at clearing *Pseudomonas aeruginosa* (52). Emerging data and data presented here suggest the existence of subpopulations of PMN with different functionality that can be polarized by their particular environment. In 2-hit scenarios, the immune system is challenged across 2 fronts. As presented here, liver trauma elicits a primary response, which — if left alone — would heal appropriately over a predictable and well-orchestrated kinetic of proinflammatory sequelae, followed by a battery of antiinflammatory responses (54). Moreover, these environments are well characterized by the presence or absence of specific cell populations that appear and disappear as damage control and healing progress. However, if a second threat occurs during the late proinflammatory or antiinflammatory phase of the initial injury, the host mounts an inappropriate response with recruitment of PMN that are designed more for damage control and healing versus an aggressive, pathogen-killing phenotype. Therefore, this heterogeneity of PMN and corresponding functionality needs to be further investigated and understood in the setting of trauma.

TLRs compose a class of evolutionarily conserved pattern recognition receptors that detect PAMPs derived from invading pathogens, in turn inducing and modulating innate immune responses to those pathogens. TLR2 recognizes Gram^+^ PAMPs like lipoprotein and peptidoglycan (34, 55, 56), and several studies have shown the importance of TLR2 in Staphylococcal infection. TLR2-deficient mice are much more susceptible to *S*. *aureus* infection than controls (34–36). Perhaps this explains why *S*. *aureus* appear more aggressive during trauma care (20). Here, mice subjected to liver crush injury were more susceptible to *S*. *aureus* infection, and this was associated with downregulation of TLR2 protein expression in BM and circulating PMN. These data suggest either that this population of PMN expressed lower TLR2 levels or that trauma and heme induce downregulation of both receptors.

In addition to TLR2, trauma and heme downregulated TLR4 in circulating PMN in mice (Supplemental Figure 3, E and F), as well as circulating PMN in trauma patients (Figure 6C). Skinner et al. also reported reductions in TLR2 and TLR4 expression in human monocytes after trauma (57). Again, this was correlated with increased risk for sepsis. One weakness of our study was the small number of patient samples. This was limited, in part, because of patient care and the need for informed consent. Our goal was to assess any overlapping findings with the mouse model in the samples available. Despite the relatively low numbers, however, we observed clear statistical significance in comparing samples from trauma patients and volunteer controls. Collectively, these data on the effects of tissue injury on TLR2 and TLR4 expression in humans and mice support a global concept that pattern recognition receptors have redundant functions and that their modulation by injury-derived DAMPs may help explain the higher rate of both Gram^–^ and Gram^+^ infections in trauma patients.

Furthermore, we observed that the levels of IP-10 and MIP-1α were significantly lower in BAL fluid in mice with lung infection after liver crush injury. Zeng et al. showed that IP-10 is a critical cytokine in antibacterial host defense in the lung and that neutralization of IP-10 not only enhanced the number of recruited PMN to the infection, but it also impaired bacterial killing and clearance in the lung (32). This is completely consistent with our observations here. PMN actively secrete MIP-1α in response to pathogens, and lack of MIP-1α increases the host’s susceptibility to bacterial infection (33, 58). The observed reduction in MIP-1α levels in the BAL of infected mice after liver crush injury or heme is consistent with the observed impairment in bacterial clearance. While we acknowledge that different components of DAMPs and PAMPs are involved in altering the extent to which various cytokines are released, these data point to heme as a major contributing factor to the observed PMN dysfunction after trauma.

While heme is an indispensable prosthetic group of many hemoproteins, free heme — especially when excessive amounts overwhelm scavenging systems — can be detrimental to the host. Historically, extravascular hemorrhage has been thought to favor infection by iron, acting as a bacterial nutrient (59–63). More recently, though, heme has been found to be a potent DAMP that can contribute to immune activation through NF-κB signaling (12). Heme is a widely accepted ligand for TLR4 (16), and more recently, it has been reported to be a potent agonist of TLR2 in astrocytes (15). To date, though, the regulation of TLR2 and TLR4 by trauma and heme in PMN is unreported. The effects may also involve TLR1 and TLR5, whose expression was also reduced. Alveolar macrophages also showed a phenotypic switch in response to trauma and likely influence the host response to bacteria.

The pathophysiology of bacterial infection following trauma injury is complex. Trauma patients are typically immunocompetent, and the term “opportunistic” usually refers to microorganisms that are normally harmless but become pathogenic when host resistance is impaired. We see here clear mechanistic evidence of how injury can create immune suppression. *S*. *aureus* typically exists as part of the host microbiome on the skin and in the upper respiratory tract. While our animal model only tested liver as the injury site and the lung as the site of infection, trauma patients present with injuries to multiple organs with a range of severity and have a wide range of infectious events occurring at multiple barrier sites (9, 64, 65). Our cohort of trauma patients developed urinary tract infections (11%), respiratory infections (33%), and — in some cases — infections at both sites (11%). While our animal model only studied lung infection, our findings strongly support that this model is a potent and generalizable research tool that is both clinically relevant and can shed light on the complicated dynamics of the immune response to infection after injury. The model of liver crush that we describe here is comparable and adapted, in part, from others in terms of methodology and severity (66, 67). Taken together our data highlight the utility of this animal model and, importantly, identify therapeutic targets including heme and TLRs that may be important targets in the care of trauma patients by reducing the heightened incidence of infection.

## Methods

### Animals

Male C57BL/6 (H-2^b^) mice (Charles River Laboratories), *Hpx^–/–^* (The Jackson Laboratory, stock no. 029380), were used at the age of 8–10 weeks. All mice were maintained in a specific pathogen–free facility.

### Human blood PMN

Human blood samples were collected from healthy volunteers, control patients, and trauma patients who were enrolled at Beth Israel Deaconess Medical Center. Control patients were enrolled from patients scheduled for elective surgical procedures. Samples from trauma patients with Injury Severity Score with a range from 14 to 75 were collected the day after admission into the hospital. Injury Severity Score is a compiled score that reflects the severity of anatomic injuries across multiple body regions. It is commonly used as a predictor of outcomes like mortality, sepsis, and organ failure. Patients with preexisting immunosuppressive conditions were excluded from the study. Signed consent was obtained from all subjects.

#### PMN isolation.

PMN were isolated fresh from peripheral blood as described by Itagaki et al. (68). Briefly, PMN were isolated from minimally heparinized whole blood by gradient centrifugation using 1-Step Polymorph (Accurate Chemical & Scientific). Isolated PMN were stored frozen in CryoStor CS10 (Stemcell) at –80°C. The frozen samples were thawed in a 37°C water bath for 2 minutes and washed with cold PBS 2 times prior to further analysis. Of note, all samples were frozen after isolation. We show in Supplemental Figure 4 that the freezing/thawing affects specific surface maker expression, but since it likely affected all cells the same, comparisons could be made. It is logistically impossible to simultaneously obtain multiple trauma PMN samples in order to study them concurrently with control patient PMN. Therefore, the best option for measuring expression was to use frozen preparations that were batched and run together in a standardized fashion.

### Liver crush injury in mice

To simulate blunt force liver trauma, a Crile needle-holder (Integra Lifesciences) was modified by adding a stem and screw to the locking ratchet. This prevents the tips from closing completely and provides reproducible compression. Mice were anesthetized with isoflurane using a calibrated vaporizer (3% v/v) during the procedure. Fifteen minutes prior to the liver crush procedure, 1.2 mg/kg of sustained release buprenorphine was administered s.c. A laparotomy was performed, and the left liver lobe was crushed 10 times uniformly. Animals were allowed to recover under controlled heat. Shams underwent laparotomy only, without liver crush.

### Bacterial lung infection and counts

*S. aureus* (ATCC) or *E*. *coli* (ATCC) was grown to saturation overnight at 37°C in 100 mL Tryptic Soy Broth (TSB; BD 25923). The next day, 2 mL of the overnight culture was grown in fresh 100 mL TSB at 37°C for 2 hours to reach log phase growth. The bacterial culture was then centrifuged at 3000*g* for 10 minutes at 4°C and diluted in sterile saline to an OD_600_ of 0.3 (or 0.6 for the survival study). To determine the number of bacteria, 10 μL of serially diluted bacterial suspensions were streaked on TSA plates (BD Biosciences, 90002) and counted the following day.

Mice (liver crush or sham controls) were anesthetized with ketamine (10 mg/kg; i.p.; distributed by Vedco. Inc., NDC 50989-161-06) and xylazine (4 mg/kg; i.p.; distributed by Covetrus North America, NDC 11695-4024-1). A small incision was made through the dermis in the neck. The sublingual glands and the sternothyroid and sternocleidomastoid were gently dissected to expose the trachea. A 30-gauge needle was carefully inserted between the cartilage rings, and 50 μL of bacterial suspension (1 × 10^6^ to 1 × 10^7^ CFU) was slowly infused during inspirations. After inoculation, the neck wound was closed with a surgical staple. Mice that reached a body condition score of 2 — which includes lethargy, lack of movement, and a drop in body temperature — were designated as moribund and, therefore, were humanely euthanized. Twenty-four hours later, mice were overdosed with anesthesia, and the heart was transected; the trachea was then intubated, and BAL was performed 3× with 1 mL of PBS. Combined BAL fluid was separated from the cell pellet by centrifugation (200*g* for 5 minutes at 4°C). The number of CFU of bacteria in the BAL fluid was measured by plating 10 μL of the fluid or serially diluted fluid on TSA plates and counting the following day. Protein levels in BAL fluid were determined using a Pierce BCA Protein Assay Kit (Thermo Fisher Scientific). Residual RBCs in the cell pellet were lysed (AKC Lysing Buffer, Thermo Fisher Scientific), and BAL leukocytes were processed for further analysis. Recovered cells were counted on a hemocytometer, and cytospin slides were prepared by cytocentrifugation onto positively charged microslides at 1800 rpm for 7 minutes and stained using Hema3 Staining Kit (Thermo Fisher Scientific). Cell differentials were analyzed by morphological criteria of 300 cells. To determine bacteria CFU in lung tissue, the lung tissue was homogenized in 1 mL of PBS and centrifuged at 200*g* for 10 minutes at 4°C, and 10 μL of the supernatant was diluted 1:50 or 1:500 and plated on TSA plates. CFU were counted the following day. For the number of CFU of bacteria in the blood, whole blood was collected by cardiac puncture in EDTA-coated tubes and the plasma was collected by centrifuging at 200*g* for 10 minutes at 4°C. In total, 50 μL of the plasma was plated on TSA plates, and CFU was counted the following day.

### Hpx treatment

Hpx (Sigma-Aldrich, SRP6514) was prepared in PBS, and mice were dosed 2 times at 50 mg/kg in 200 μL immediately after (i.v.) and again 45 minutes after liver crush injury (i.p.). Control mice received PBS in the same volume and at the same intervals.

### Heme challenge

Heme (Sigma-Aldrich, 52180) was prepared by adjusting the pH to 6.9–7.2 using 1N NaOH (Sigma-Aldrich) and 6N HCl (Sigma-Aldrich) solutions in PBS and then to a final concentration of 5 mg/mL in PBS. Mice were dosed at 50 mg/kg, i.p.

### ALT, heme, and Hpx measurements

Whole blood was collected by cardiac puncture in EDTA-coated tubes. Plasma was collected by centrifugation at 3000*g* at 4°C for 15 minutes. Plasma was kept on ice or at –20°C for longer storage. ALT in the plasma samples was measured by using an IDEXX Catalyst DX analyzer (IDEXX Laboratories). Heme concentration in plasma was measured by the hemin colorimetric assay kit (GWB-AXR320, Genway Biotech). Hpx in plasma was measured by ELISA (NBP2-60633, Novus Biologicals).

### PMN isolation from BM and ROS measurement

Femurs were removed from mice, and BM cells were collected by centrifugation (13,000*g* for 15 minutes at 4°C). RBCs were lysed (ACK Lysing Buffer, Thermo Fisher Scientific). BM cells were washed with PBS, and PMN was isolated using Miltenyi’s Neutrophil Isolation Kit (130-097-658, Miltenyi Biotec). In total, 1.25 × 10^6^ PMN were incubated in 10% RPMI + 10% FBS and 90% naive or trauma serum for 90 minutes. Trauma serum was prepared from whole blood 30 minutes after liver crush injury. After incubation with serum, PMN were washed with PBS, and ROS release was measured for 2 hours after being challenged with 1 × 10^6^ CFU of *S*. *aureus*. ROS generation was performed as described by Konecna et al. (69).

### Histologic analyses

The left liver lobe was collected from mice with or without liver crush injury, and 5 μm sections were stained with H&E. Lung tissue was collected 24 hours after infection with or without prior liver crush injury. The left lung was fixed by instilling 500 μL of 10% buffered formalin. After processing, 5 μm sections were stained with H&E and blindly evaluated for inflammatory cells, hemorrhage, edema and changes in architecture.

### Gene expression

Total RNA was extracted from tissues using TRizol reagent (Invitrogen) following the manufacturer’s instruction. Reverse transcription was performed using High Capacity cDNA synthesis kit (Thermo Fisher Scientific). After cDNA synthesis, quantitative PCR (qPCR) was performed on QuantStudio 3 (Applied Biosystems) using Maxima SYBR Green Master Mix (Thermo Fisher Scientific).

For samples from mice, the following primers (written in a 5**′**-3**′** direction) were used: *Actb* (F: CCTTCTTGGGTATGGAATCCTGT, R: GAGGTCTTTACGGATGTCAACG), *Hmox1* (F: CAGAAGAGGCTAAGACCGCC, R: AGCTCCTCAAACAGCTCAATGT), *Hprt1* (F: CAGTCCCAGCGTCGTGATT, R: GCAAGTCTTTCAGTCCTGTCCAT), and *Tlr2* (F: ACCTCAGACAAAGCGTCAAA, R: ACAGCGTTTGCTGAAGAGGA).

Fold-change in mRNA expression was normalized to *Actb* or *Hprt1* by using the ΔΔCt method.

### Immunoblotting

In total, 200 mg of the injured liver lobes, uninjured liver lobes, and lungs from the euthanized mice were homogenized in RIPA lysis buffer containing halt protease inhibitors (100:1) at 4°C. The tissue homogenates were vortexed every 5 minutes for 30 minutes on ice and centrifuged at 12,000*g* for 15 minutes at 4°C. The supernatant containing the tissue protein was collected, and the concentration of protein was determined by the Pierce BCA Protein Assay Kit (Thermo Fisher Scientific). Equal amounts of proteins (20 or 30 μg) were loaded and separated by 4%–15% polyacrylamide gel (Bio-Rad) and transferred onto nitrocellulose membranes at 110 mA and 18V for 70 minutes. Membranes were blocked with 5% nonfat milk (Nestle) in tris-buffered saline (TBS; Thermo Fisher Scientific, 77-86-1) and Tween 20 (TBS-T; Boston BioProducts, P-934) buffer overnight at 4°C with gentle rocking. The membranes were then incubated with primary antibody against HO-1 (1:1000, ab52947, Abcam) and vinculin (1:1000, MAB6896, R&D Systems) for 2 hours at room temperature with gentle rocking. The membranes were washed with TBS-T buffer for 5 minutes for 3 times and then with TBS buffer for 5 minutes with gentle rocking at room temperature. Then, the membranes were incubated with IRDye 680RD goat antirabbit (1:15000, LI-COR, 925-68071) or IRDye 800RD goat anti-rabbit (1:15000, LI-COR, 800925-32211) for 90 minutes at room temperature and then washed with TBS-T buffer for 5 minutes for 3 times and with TBS buffer for 5 minutes. The images were visualized on the Odyssey CLx Imaging System, and the band intensities were quantified using Image Studio Lite software and normalized to vinculin as a loading control.

### Flow cytometry

#### Mouse.

Peripheral blood was collected by cardiac puncture into EDTA tubes. BM cells were isolated by centrifuging femurs (13,000*g* for 15 minutes at 4°C). Liver tissues were homogenized gently using a 3 mL syringe plunger. RBCs were lysed (ACK Lysing Buffer, Thermo Fisher Scientific). Any clumps of cells were removed by filtration using 70 μm strainers (Thermo Fisher Scientific). Cells were washed and kept in PBS supplemented with 2% FBS and were stained with the following fluorochrome-conjugated antibodies: CD45 (Alexa Fluor 700, BioLegend, 103128, 1:300), CD11b (Brilliant Violet 421, BioLegend, 101235, 1:500), Ly6G (APC, BioLegend, 127614, 1:300; FITC, BioLegend, 127605, 1:500), CD11c (Alexa Fluor 488, BioLegend, 117313, 1:100; PE-Cyanine7, BioLegend, 117318, 1:100), TLR2 (PE, BioLegend, 148604, 1:200), TLR4 (PE-Cyanine7, BioLegend, 117610, 1:200), TLR1 (PE, Thermo Fisher Scientific, 12-9011-80, 1:100), and TLR5 (APC, BioLegend, 148105, 1:100) at 4°C and washed twice. Intracellular staining for HO-1 was performed by permeabilizing the cells using the BD Cytofix/Cytoperm Fixation/Permeabilization Kit (BD Biosciences) and staining the cells with antibody against HO-1 (ab52947, 1:200), followed by donkey anti-rabbit secondary antibody (BioLegend, 406414, 1:500).

#### Human.

Cells were stained with the following fluorochrome-conjugated antibodies: TLR2 (APC, BioLegend, 309719, 1:100; FITC, BioLegend, 309705, 1:100), TLR4 (PE, BioLegend, 312805, 1:100), CD15 (FITC, BioLegend, 301903, 1:100), CD16 (Alexa Fluor 700, BioLegend, 302025, 1:100), CD11b (Brilliant Violet 421, BioLegend, 101235, 1:100), CD66b (PE-Cyanine7, BioLegend, 305115, 1:100). The stained cells were run on CytoFLEX Flow Cytometer (Beckman Coulter). Flow cytometry data were analyzed by FlowJo software. Flow cytometry gating strategy is shown in Supplemental Figure 5.

### Cytokine/chemokine determination

BAL fluid was analyzed on a 32-plex array by Eve Technologies.

### Statistics

All data represent mean ± SEM. Data were compared by unpaired 2-tailed Student’s *t* test, or by 1-way ANOVA with post hoc Tukey’s test using GraphPad Prism 8. A *P* value less than 0.05 was considered significant.

### Study approval

#### Animal studies.

All procedures involving animals were approved by the Beth Israel Deaconess Medical Center IACUC and the Department of Defense, USAMRMC Animal Care and Use Review Office.

#### Human studies.

The Code of Ethics of the World Medical Association (Declaration of Helsinki) was followed, and the study was approved by the IRB of Beth Israel Deaconess Medical Center. A written informed consent form was obtained from all individuals.

## Author contributions

GRL, DG, and LEO conceived and designed the study. GRL, DG, RWADS, STH, and EC acquired data. JDH and SS acquired and processed human samples. GRL and VBG analyzed clinical data. GRL analyzed data. GRL and LEO wrote the paper. RWADS, MBY, MSL, and CJH provided critical revision of the paper.

## Supplementary Material

Supplemental data

## Figures and Tables

**Table 1:**
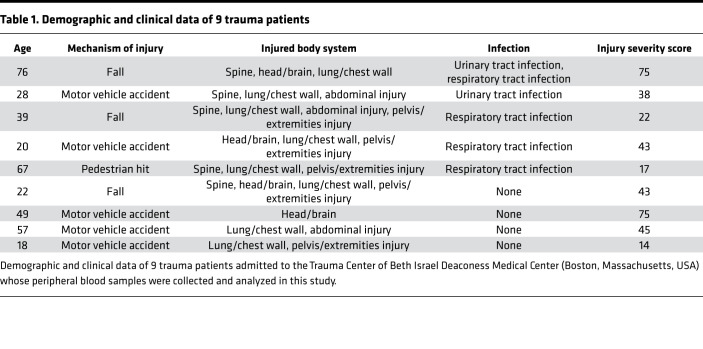
Demographic and clinical data of 9 trauma patients

## Version 1 09/14/2021

In-Press Preview

## Version 2 10/22/2021

Electronic publication
