# Supplementary material for: Trauma-induced heme release increases susceptibility to bacterial infection
Source: JCI Insight. 2021 Oct 22;6(20):e150813. doi: 10.1172/jci.insight.150813 (PMC8564912; doi:10.1172/jci.insight.150813)
Supplement: Supplemental data [file jciinsight-6-150813-s199.pdf]

Supplemental Figure 1

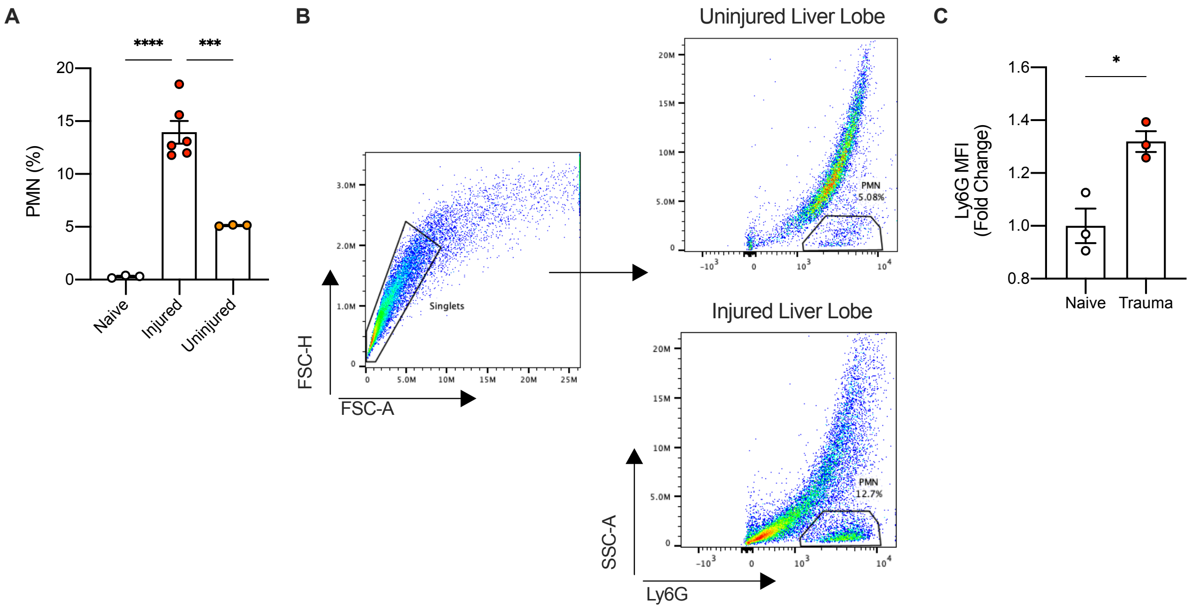

Supplemental Figure 1. PMN infiltrate to the liver after liver crush injury. (A) Frequency of PMN in naive, injured left liver lobe, and non-injured right liver lobe, 4 hours after liver crush injury (n=3-6/group). **(B)** Representative flow cytometry analysis images are shown. Singlets are gated by FSC-A vs FSC-H and a population of $Ly6G^{+}$ PMN was identified (top: uninjured liver lobe; bottom: injured liver lobe). **(C)** Fold change of Ly6G MFI in circulating PMN 4 hours after liver crush injury (n=3/group). All data are presented as mean ± SEM. Statistical analysis was performed by using unpaired Student's *t* test or one-way ANOVA with post-hoc Turkey's test. *P<0.05, ***P<0.001, ****P<0.0001

Supplemental Figure 2

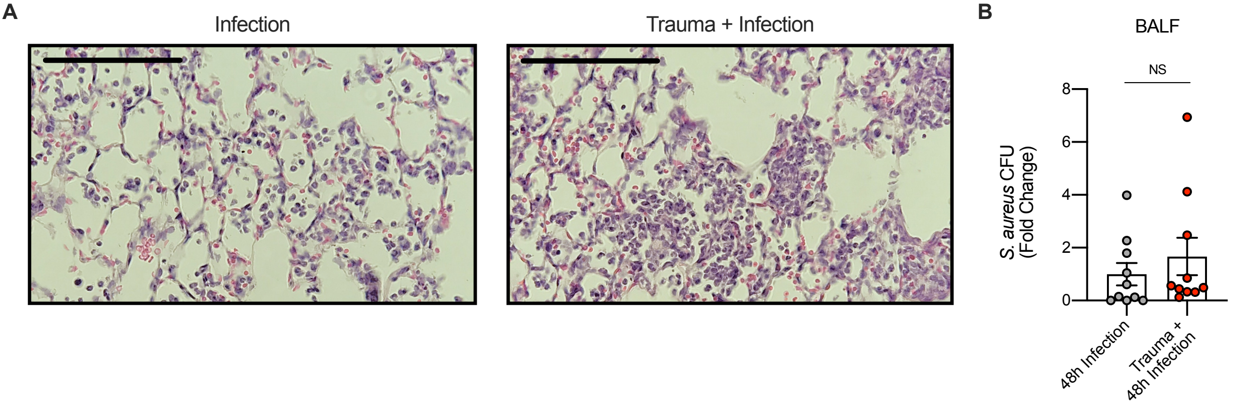

Supplemental Figure 2. (A) Representative images of the infected lung with (right) and without liver crush injury (left) from 3 animals with 6 sections taken from each lung. Slides were evaluated blinded. Scale bar = 100µm. Original magnification, x200. **(B)** Fold change of *S. aureus* CFU in BALF of mice with and without liver crush injury. BALF was collected 48 hours after infection (n=10/group). Statistical analysis was performed by using unpaired Student's *t* test. All data are presented as mean ± SEM. NS = Not Significant

Supplemental Figure 3

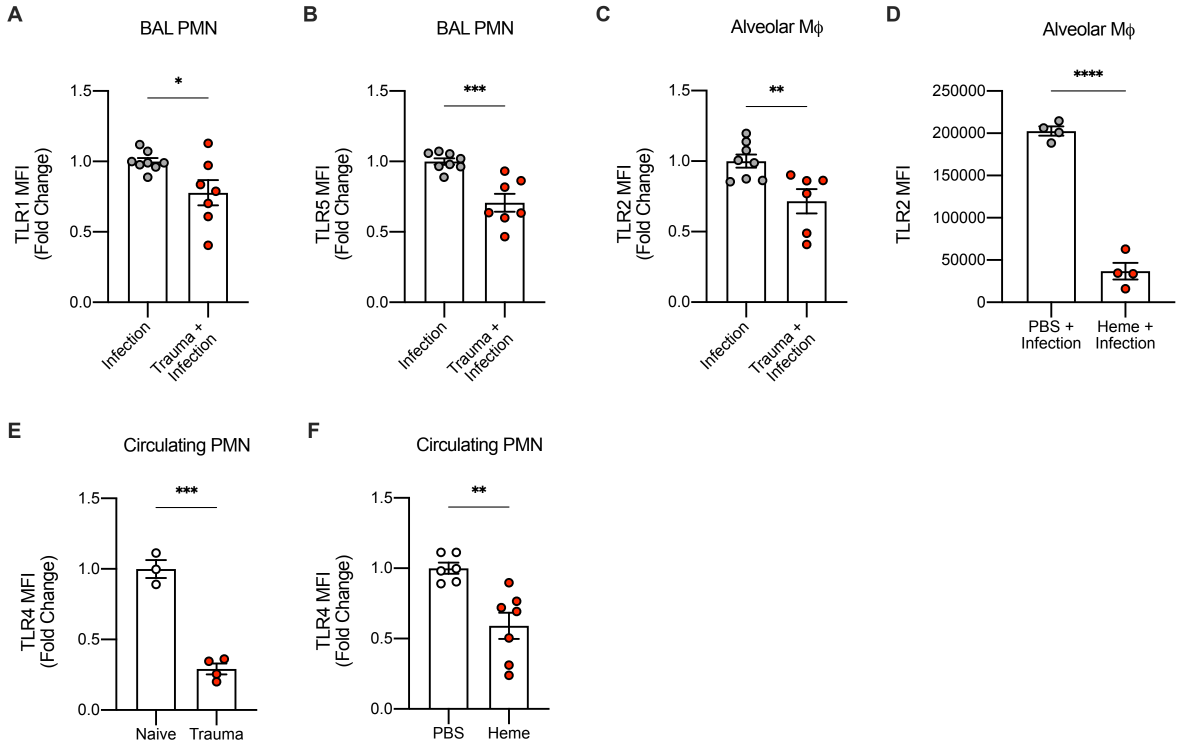

Supplemental Figure 3. Changes in TLR1, TLR2, TLR4 and TLR5 expression after liver crush injury or heme challenge. (A-B) Fold change of TLR1 and TLR5 MFI in BAL PMN 24 hours after infection with and without liver crush injury (n=7-8/group). **(C)** Fold change of TLR2 MFI in alveolar macrophages 24 hours after infection with and without liver crush injury (n=6-8/group). **(D)** TLR2 MFI in alveolar macrophages 24 hours after infection with and without heme challenge (50mg/kg, i.p., n=4/group. **(E)** TLR4 MFI in circulating PMN 4 hours after liver crush injury (n=3-4/group). **(F)** TLR4 MFI in circulating PMN 4 hours after heme challenge (50mg/kg, i.p., n=6-7/group). All data are presented as mean ± SEM. Statistical analysis was performed by using unpaired Student's *t* test. *P<0.05, **P<0.01, ***P<0.001, ****P<0.0001, NS = Not Significant

Supplemental Figure 4

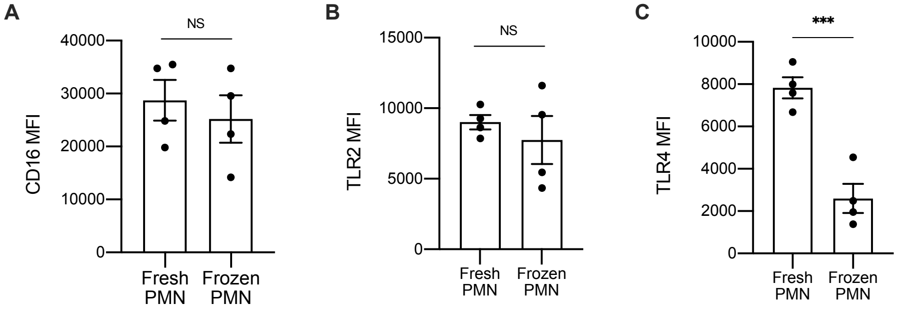

Supplemental Figure 4. Changes in the surface markers of human PMN before and after a cycle of freezing and thawing. (A-C) MFI of CD16, TLR2, and TLR4 in fresh PMN and the same PMN that have been thawed after having been frozen for 24 hours (n=4/group). All data are presented as mean ± SEM. Statistical analysis was performed by using unpaired Student's *t* test. ***P<0.001, NS = Not Significant

Supplemental Figure 5

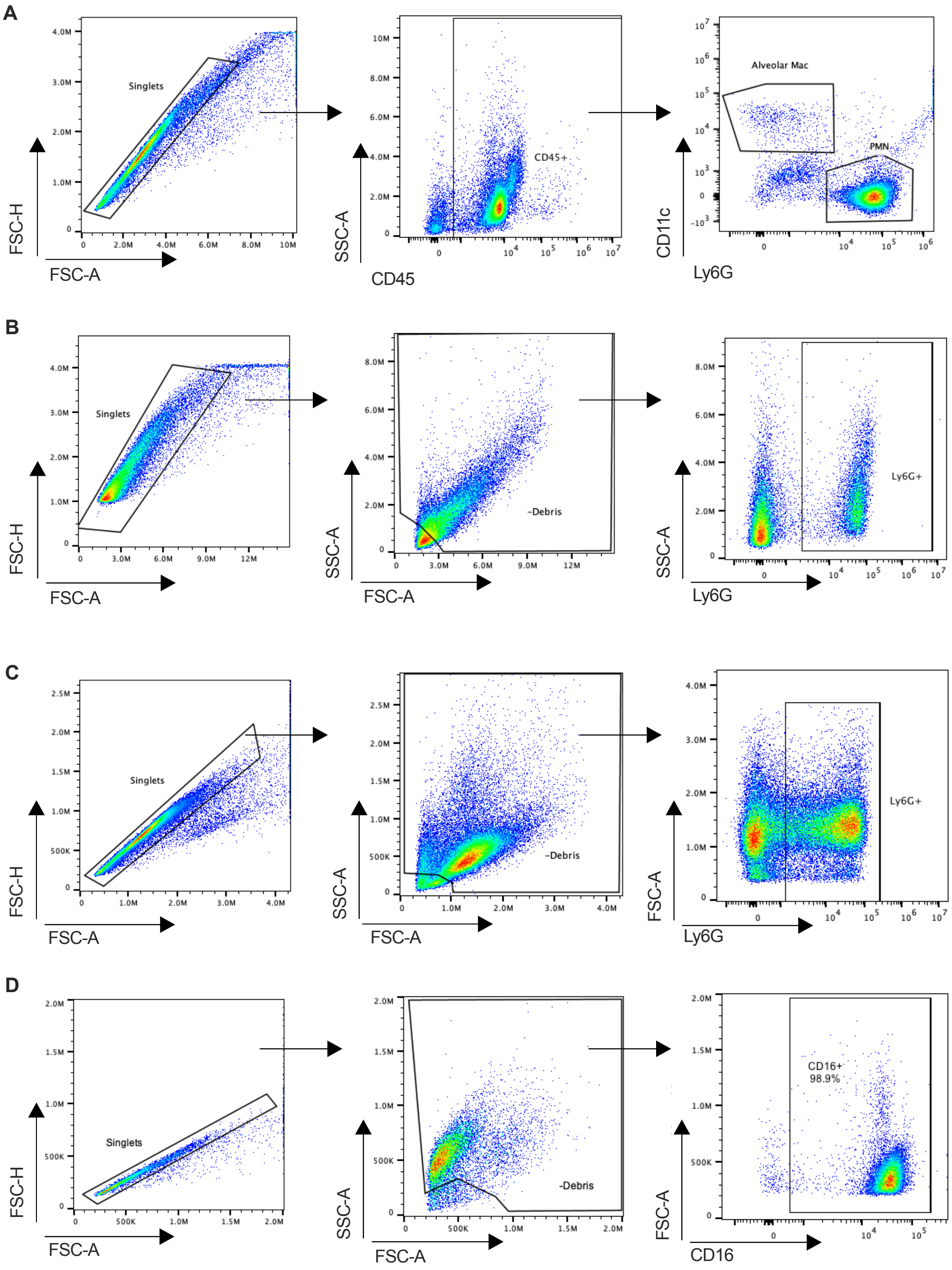

Supplemental Figure 5. Flow cytometry gating strategy. (A-C) Representative flow cytometry gating images for BAL, blood cells, and bone marrow cells, respectively, in mice. **(D)** A representative flow cytometry gating image for circulating PMN purified from the whole blood in human.
